# Characterization of positioning uncertainties in PET‐CT‐MR trimodality solutions for radiotherapy

**DOI:** 10.1002/acm2.13617

**Published:** 2022-04-28

**Authors:** Pauline Hinault, Isabelle Gardin, Pierrick Gouel, Pierre Decazes, Sebastien Thureau, Ovidiu Veresezan, Henri Souchay, Pierre Vera, David Gensanne

**Affiliations:** ^1^ QuantIF‐LITIS EA4108 University of Rouen Normandie Rouen France; ^2^ GE Healthcare Buc France; ^3^ Nuclear Medicine Department Henri Becquerel Cancer Center Rouen France; ^4^ Radiotherapy Department Henri Becquerel Cancer Center Rouen France

**Keywords:** multimodality imaging, positioning, radiotherapy

## Abstract

The purpose of this study was to evaluate the positioning uncertainties of two PET/CT‐MR imaging setups, C1 and C2. Because the PET/CT data were acquired on the same hybrid device with automatic image registration, experiments were conducted using CT‐MRI data. In C1, a transfer table was used, which allowed the patient to move from one imager to another while maintaining the same position. In C2, the patient stood up and was positioned in the same radiotherapy treatment position on each imager. The two setups provided a set of PET/CT and MR images. The accuracy of the registration software was evaluated on the CT‐MRI data of one patient using known translations and rotations of MRI data. The uncertainties on the two setups were estimated using a phantom and a cohort of 30 patients. The accuracy of the positioning uncertainties was evaluated using descriptive statistics and a *t*‐test to determine whether the mean shift significantly deviated from zero (*p* < 0.05) for each setup. The maximum registration errors were less than 0.97 mm and 0.6° for CT‐MRI registration. On the phantom, the mean total uncertainties were less than 2.74 mm and 1.68° for C1 and 1.53 mm and 0.33° for C2. For C1, the *t*‐test showed that the displacements along the z‐axis did not significantly deviate from zero (*p *= 0.093). For C2, significant deviations from zero were present for anterior‐posterior and superior‐inferior displacements. The mean total uncertainties were less than 4 mm and 0.42° for C1 and less than 1.39 mm and 0.27° for C2 in the patients. Furthermore, the *t*‐test showed significant deviations from zero for C1 on the anterior‐posterior and roll sides. For C2, there was a significant deviation from zero for the left‐right displacements.This study shows that transfer tables require careful evaluation before use in radiotherapy.

## 1 INTRODUCTION

Radiotherapy aims to ensure local tumor control while preserving healthy tissues. A high degree of spatial accuracy is required to delineate the gross tumor volume (GTV) and position the patient during preparation and treatment. For instance, in the case of head and neck (H&N) cancers, this accuracy can reach 1–2 mm with adapted restraints such as thermoformed masks.^1^ Computed tomography (CT) is a reference imaging modality in radiotherapy treatment planning, which is used by radiation oncologists to delineate volumes of interest^2^ and for mapping of the electron tissue densities, which is necessary for dosimetry treatment planning. CT is known to ensure excellent spatial accuracy; however, it may present several limitations. For example, metallic implants^3^ can cause difficulties in the delineation of H&N tumors. In addition, the low contrast between soft tissues^4^ adds complexity to the delineation of certain tumoral lesions. Therefore, other imaging modalities may be used to complement the information provided by CT.

Magnetic resonance imaging (MRI) is another modality of interest in radiotherapy, especially for defining certain volumes such as prostatic^5^, ^6^ and H&N tumors.^7^, ^8^, ^9^ However, the substitution of CT with MRI remains limited by the lack of information on tissue electron density and the presence of geometric distortions. Several authors have proposed methods of converting MRI to CT images, corresponding to pseudo‐CT images; however, there is currently no consensus between these methods, and this area is still in development.^10^

Positron emission tomography (PET) with F‐18‐fluorodeoxyglucose (FDG) is also important in cancer patient management.^11^ The integration of FDG‐PET images in the segmentation of GTVs has been widely proposed in literature.^12^ Most PET devices are used with CT, which improves the reproducibility of target volume segmentation.^13^ A significant reduction in the inter‐observer variability of tumor delineation has been observed when PET was used alongside CT compared with CT alone (*p* < 0.001).^14^

Trimodality imaging (PET, CT, and MRI) has improved the definition of GTVs.^15^, ^16^, ^17^, ^18^, ^19^, ^20^ However, no single medical device allows the acquisition of all three imaging modalities. The most widespread solution consists of using a hybrid imager coupled with a second independent imager, for example, PET/CT and MRI (PET/CT‐MRI). It is also possible to use a hybrid PET/MRI device and CT (PET/MRI‐CT). In either case, the images must be acquired in the radiotherapy treatment position on a specific bed adapted to the two imaging devices that are compatible with the specific equipment for radiotherapy treatment (that is, a rigid tabletop and immobilization fixations, such as a thermoformed mask and head and knee wedges for H&N tumors). Generally, after PET/CT‐MRI data acquisition, image registration is automatic because the PET/CT data are acquired on the same hybrid device.^21^, ^22^ However, CT‐MRI image registration is necessary,^23^ leading to the first source of uncertainty in patient positioning.

In most cases, the patient is positioned on each imaging device, PET/CT and MRI, under the same conditions as in radiotherapy treatment. The advantage of this method is that the images can be acquired on the first device and then on the second device in the following days; the patient does not have to undergo two examinations on the same day. However, despite its benefits for image registration, this method is possibly detrimental to the quality of the patient positioning.

Another less‐developed approach consists of transporting the patient, positioned in the same conditions as in radiotherapy treatment, from one imager to the other on a table compatible with the two acquisition devices. This strategy prevents the patient from getting up.^24^, ^25^ The advantage of this approach is a potential improvement in patient positioning; however, acquisitions between the two imaging devices must be synchronized. This second solution has already been studied in the context of PET/CT‐MRI trimodality,^24^ although only translations have been evaluated. These were between 5 and 8 mm in the x‐and y‐directions. This transfer solution was also presented as part of an offline MRI‐guided radiotherapy solution.^26^ Patients were moved from the MRI to the treatment machine using an air‐cushioned bed transfer system. The difference in position between the MRI, on‐board imaging, and planning CT was evaluated for each treatment fraction, and the total translation uncertainty was evaluated as less than 2 mm.

The main objective of this study was to evaluate the positioning uncertainties in both translation and rotation related to trimodality imaging for radiotherapy with two setups of a PET/CT‐MRI solution. The first setup, denoted as C1, used a transfer table, which allowed the patient to switch from one imager to another while maintaining the same position. The second, denoted as C2, consisted of positioning the patient in an identical radiotherapy treatment position on each imager. Firstly, the accuracy of the image registration software was evaluated using known induced translation rotations of the patient's data. Secondly, the uncertainties on the two setups were estimated using a phantom by analyzing the rigid transformation matrix that was used to register the CT‐MR images. Finally, the same workflow was used to determine the positioning uncertainties for a cohort of 30 patients treated with radiotherapy for H&N tumors.

## 2 METHODS

### 2.1 Imaging devices and transfer system

Two imaging devices were used in this study: a PET/CT Discovery 710® and Optima MR 450w® (GE Healthcare, IL, USA). Both devices were equipped with external lasers, similar to those in the radiotherapy treatment room. The imaging devices were located on the same floor of the same institution along a 40 m path, without obstacles (steps).

Auxiliary equipment was used to perform acquisitions during radiotherapy treatment. A rigid flat top was positioned on the table of each device, and these two flat tops were PET/CT‐and MRI‐compatible (DIACOR, UT, USA). Both had an indexing system to fix the radiotherapy restraints. All of these devices and equipment correspond to C2.

**FIGURE 1:**
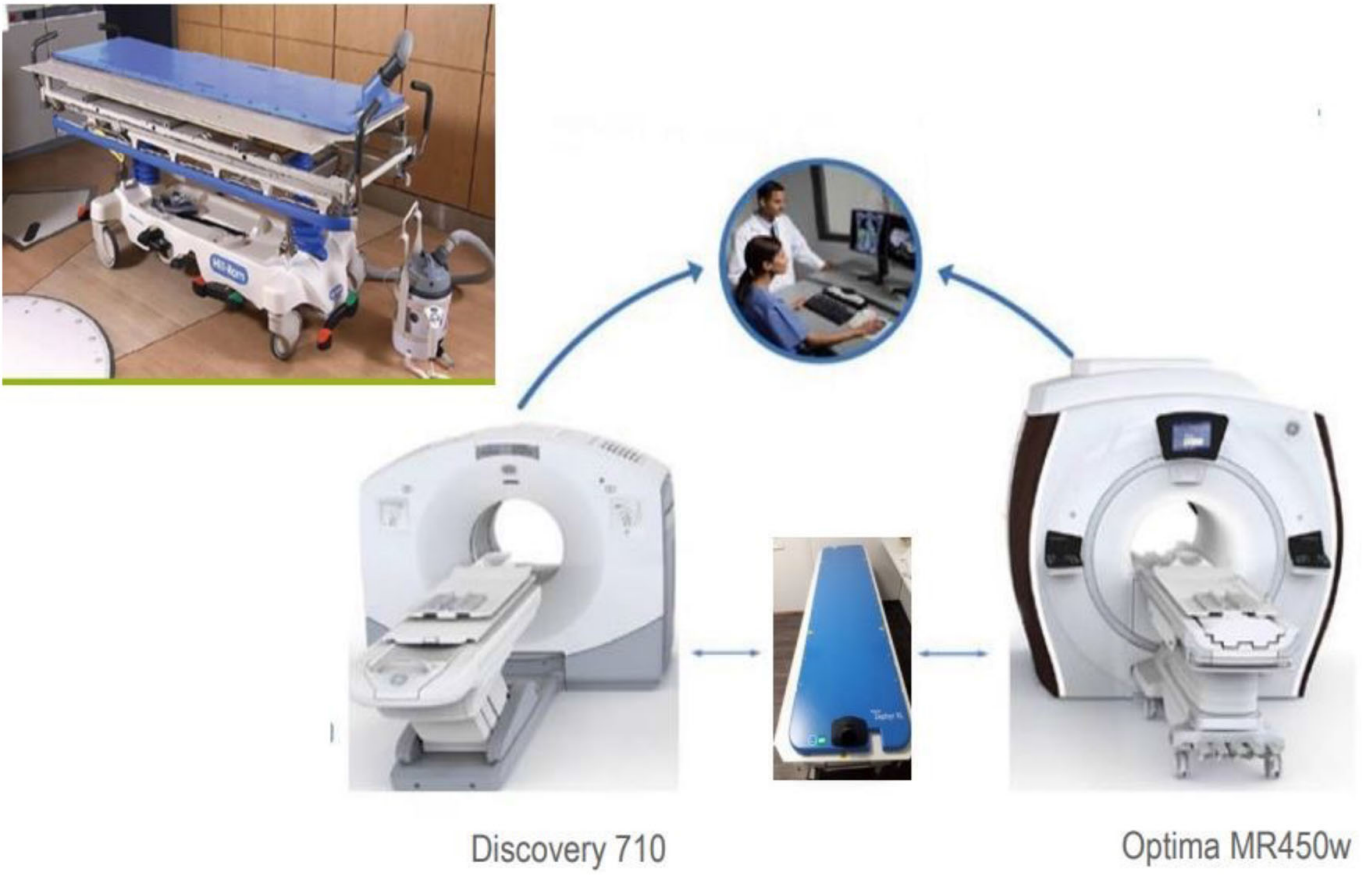
PET/CT‐MRI trimodality system

Both imaging devices were compatible with a transfer system (DIACOR) (see Figure 1), which was composed of an air‐cushion bed with low attenuation (Zephyr XL®) and a nonmagnetic stretcher (Zephyr MR Conditional Stretcher), allowing the bed to be moved and used from one device to another. These devices and equipment correspond to C1. The air‐cushioned bed was placed on the flat top of the first device, and the patient was placed in the position that he or she would be in during each radiotherapy treatment session, that is, with a thermoformed mask and knee wedge. At the end of the acquisition, the patient remained lying down, and the air‐cushioned bed was moved to the stretcher using a suction system. The patient was transferred to the second imaging device, and the air bed was moved from the stretcher to the flat top of the device table using the same suction system. Finally, the C1 allowed multimodal acquisitions while maintaining the patient in the same position.

In each experiment, there were two PET/CT‐MRI datasets per setup, and because the PET/CT data were acquired using the same hybrid device, they were registered automatically by the device. The data used included only the CT and MRI data.

### 2.2 Phantom

**FIGURE 2:**
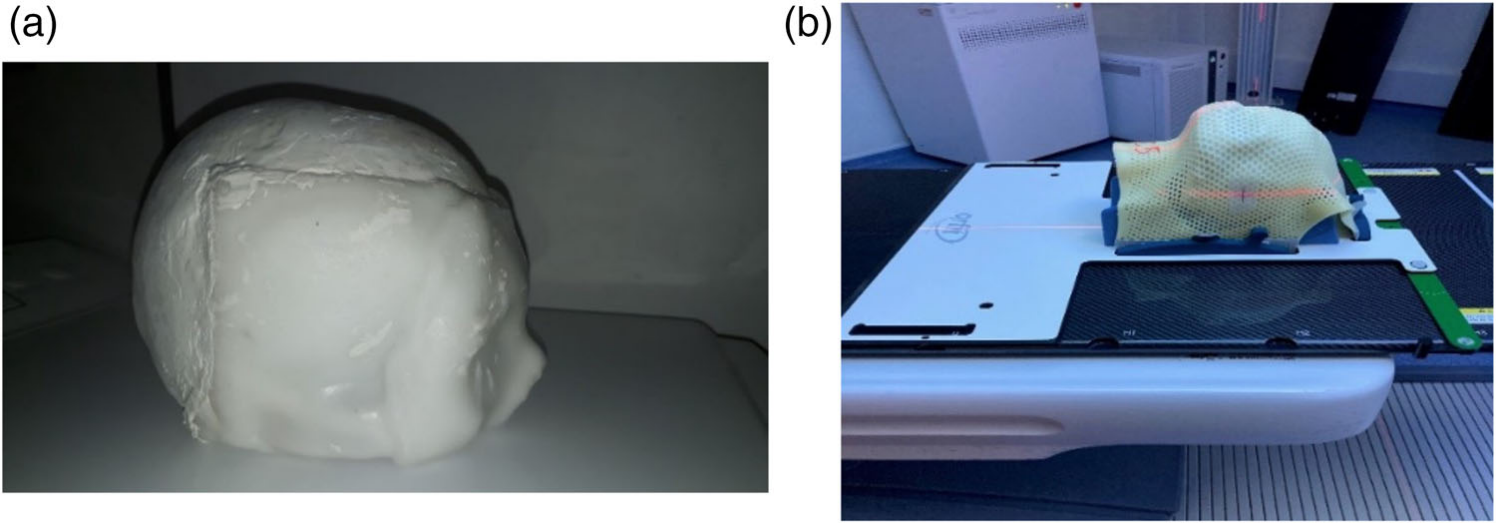
(a) Homemade ceramic skull phantom filled with gelatin. (b) Complete phantom with a thermoplastic mask positioned on the rigid tabletop with positioning lasers visible on the side (red lines on the phantom mask)

A homemade phantom was designed to evaluate the positioning uncertainties associated with the trimodality technique independent of patient motion. It consisted of a ceramic skull with a volume of 1500 cc (see Figure 2a). To obtain a rigid material that mimics the physical properties of tissues for both CT and MRI, the phantom was composed of water and 10% swine gelatin. A thermoplastic mask and suitable head wedge were used to image the phantom under radiotherapy treatment conditions (see Figure 2b).

### 2.3 Accuracy of image registration

The BODY algorithm implemented on the AW Server® module (Version 3.2 (GE Healthcare)) was used to register multimodal images. This algorithm is based on rigid registration using mutual information.^27^ For this study, registration was performed on a dataset that corresponds to the CT‐MRI images of a patient treated for H&N cancer.

The patient was randomly selected from a database used later (see Section 2.4.2), and MR images were acquired using a GEM RT Head® (GE Healthcare) radiotherapy coil configuration. The images obtained were those of a 3D CUBE T1 sequence, and the acquisition parameters were as follows: TE = 13.7 ms/TR = 352 ms, FOV = 448 mm, a 448 × 448 matrix, and a slice thickness of 2.5 mm. For the CT data, the acquisition parameters were 120 kVp, 397 mA, a revolution time of 0.8 s, pitch of 0.94, reconstruction diameter of 500 mm, matrix of 512 × 512, and slice thickness of 2.5 mm. CT images were reconstructed using an OSEM (Ordered Subset Expectation Maximization) iterative algorithm (two iterations, 24 subsets). The spatial resolutions were of 1 mm for MRI and 1 mm for CT.

For PET/CT and MRI acquisitions, the patient was placed in the same position as during each radiotherapy treatment session. He was placed on an appropriate flat top with a knee wedge and a personalized thermoformed mask (HP Efficast Orfit) fixed to a support (MRI‐G Orfit) that was rigidly attached to the hard plane. Using the BODY algorithm, an initial automatic registration was performed between the CT and MR images.

Known translations were then introduced on the MRI dataset in each direction of space (*x,y,z*). The images were modified by translations ranging from 0.5 to 20 mm in increments of 1 mm. Similarly, the orientation of the MR images was modified with rotations (pitch, roll, and yaw) from 0.2° to 5° and a pitch of 0.2°.

**FIGURE 3:**
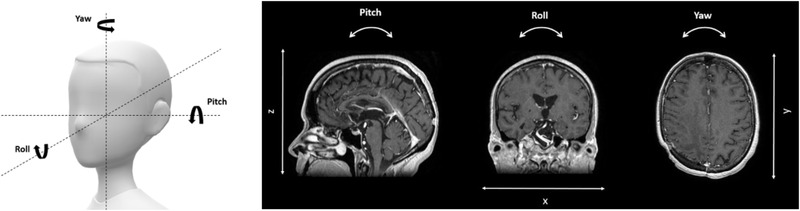
Translation and rotation transformations

These two types of transformations were independently studied. In each experiment, the BODY algorithm was used to register the modified MR images on the CT images, and differences between the expected and actual registrations were evaluated. The translation values obtained via the transformation matrix were directly compared to the induced translations. Additionally, the rotations were calculated by solving the 3D rotation matrix using the registration algorithm (see Equation (1)). The angle *ϕ* corresponds to the pitch, *θ* corresponds to the roll, and *α* to the yaw (see Figure 3). For each transformation, the image registration process was repeated ten times.

(1) $$Rx,y,z\left(\varphi ,\theta ,\alpha \right)=\left(\begin{array}{ccc} \cos\theta \cos\alpha & -\cos\theta \sin\alpha & \sin\theta \\ \cos\alpha \sin\varphi \sin\theta +\cos\varphi \sin\alpha & -\sin\alpha \sin\varphi \sin\theta +\cos\varphi \cos\alpha & -\sin\varphi \cos\theta \\ -\cos\alpha \cos\varphi \sin\theta +\sin\varphi \sin\alpha & \cos\varphi \cos\theta \sin\alpha +\sin\varphi \cos\alpha & \cos\varphi \cos\theta \end{array}\right)$$

### 2.4 CT‐MRI registration shifts relative to the trimodality techniques

#### 2.4.1 Phantom study

This study contained two experiments: one in which the transfer bed was used (C1) and the other in which it was not (C2). For C1, the phantom was positioned on the MRI device and then transferred to the PET/CT device with the transfer bed. For C2, the phantom was first positioned on the MRI device and then positioned under the same conditions on the PET/CT device. The markings on the phantom mask were centered using positioning lasers to maintain the same position for both MRI and PET/CT. The remainder of the experiments were identical in both cases. The phantom was positioned under conditions similar to those in radiotherapy, using a thermoformed mask fixed to a support that was rigidly attached to a rigid flap top.

All MR images were acquired using dedicated radiotherapy coils (GEM RT Head®, GE Healthcare), allowing acquisition in the treatment position. For MRI and CT, the acquisition conditions were the same as previously described. Each experiment was repeated ten times.

The MR and CT images were registered using a previously evaluated rigid algorithm (the BODY algorithm), leading to a 3D transformation matrix. Solving this matrix allowed us to obtain the x, y, and z translation values as well as the pitch (*θ*), roll (*ϕ*), and yaw (*α*) rotation values applied during the registration process.

The obtained values (mean positioning error, mPE) were then added in quadrature to the mean errors inherent to the registration algorithm (mRE) to obtain the total error (TE) (see Equation (2)) between the CT and MRI images.

(2) $$\mathrm{TE}=\sqrt{mRE^{2}+mPE^{2}}$$

#### 2.4.2 Patient study

This study was based on a prospective cohort of 30 H&N cancer patients treated with radiotherapy. All patients were included in a clinical trial protocol (NCT03897166) in which MRI and PET/CT were performed during radiotherapy treatment.

Half of the patients underwent the first experiment using the transfer system (C1). Therefore, they did not move between the two examinations; they underwent MRI and PET/CT while maintaining the same position. The centering for each examination was performed using markings on the mask and positioning lasers.

The other half of the patients participated in the second experiment in which the transfer bed was not used (C2); hence, they moved from one room to the other. First, the patient was positioned on MRI device and centered using markings on the mask and the positioning lasers. Subsequently, the patient stood up and was positioned on the PET/CT scanner. The markings on the mask and the positioning lasers made it possible to center the patient in the same way as in MRI.

The remaining examinations were performed under identical positioning conditions for each experiment. The patients were placed in the radiotherapy treatment position with a thermoformed mask as well as blocks and wedges placed in accordance with the degree of flexion required for a comfortable position. The second wedge was positioned under the knees. MRI and CT images were acquired and reconstructed under the same conditions as those used in the phantom experiment. Similarly, MR images were registered to the CT using the previously evaluated registration algorithm, leading to a 3D transformation matrix. By solving Equation (1), the translation and rotation values applied during the registration process can be obtained. The total errors between CT and MRI images were obtained in the same way as described above (Equation (2)).

### 2.5 Statistics

The accuracy of the registration algorithm was evaluated using descriptive statistics and Bland–Altman analysis. The descriptive statistical analysis focused on the difference in translation and rotation between those performed by the simulation and those performed by the image registration software for the CT‐MRI registrations. The analysis grouped the translations in the *xy*‐plane (*n* = 20) and along the *z*‐axis (*n* = 10), and also grouped the rotation results (*n* = 30).

Descriptive statistical analyses were performed for the phantom and patient studies to evaluate CT‐MRI registration shifts relative to the trimodality techniques. For the phantom study, a descriptive statistical analysis (*n* = 10) was performed on the two configurations to evaluate the translational and rotational shifts caused by the registration algorithm on the CT‐MR images. A one‐sample *t*‐test was used to determine whether the mean shifts significantly differed from zero (*p* < 0.05) for each configuration. For the study of patient data, a descriptive static analysis (*n* = 15) was performed on the two configurations to evaluate the translational and rotational shifts caused by the registration algorithm on the CT‐MR images. The same one‐sample *t*‐test was used once again.

## 3 RESULTS

### 3.1 Accuracy of image registration

**FIGURE 4:**
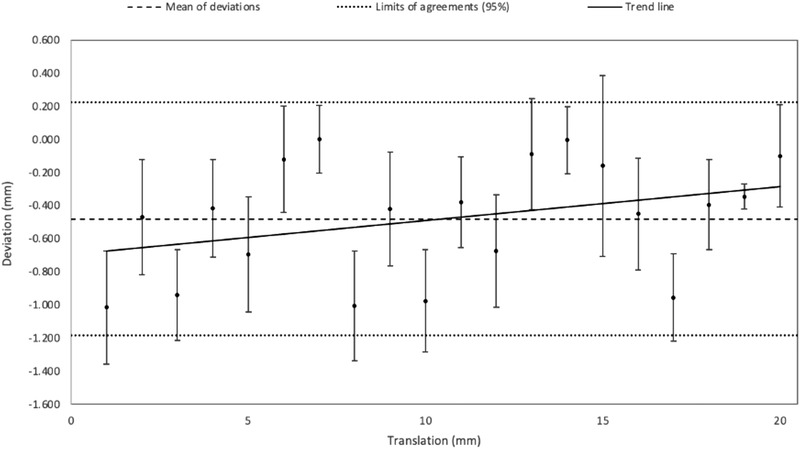
Bland–Altman graph representing the mean of the differences and standard deviation, in mm (*n* = 20, 10 in *x*, 10 in *y*), between the translations simulated on the MRI patient data and the translations operated by the registration software

**FIGURE 5:**
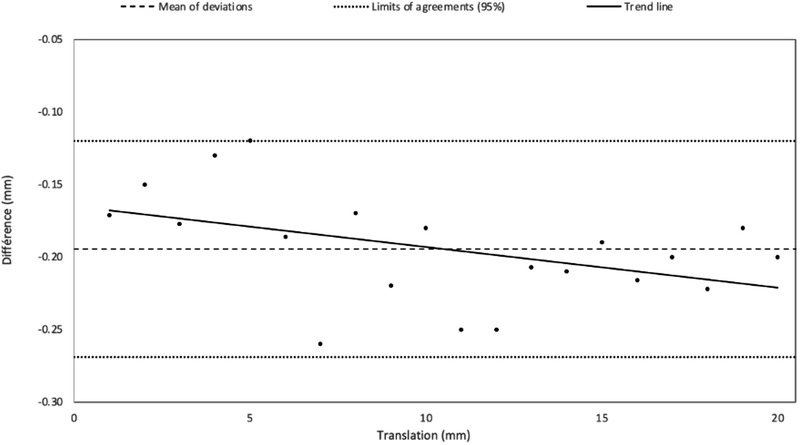
Bland–Altman graph representing the mean of the differences and standard deviation, in mm (*n* = 10), between the translations simulated in *z* on the MRI patient data and the translations operated by the registration software

**FIGURE 6:**
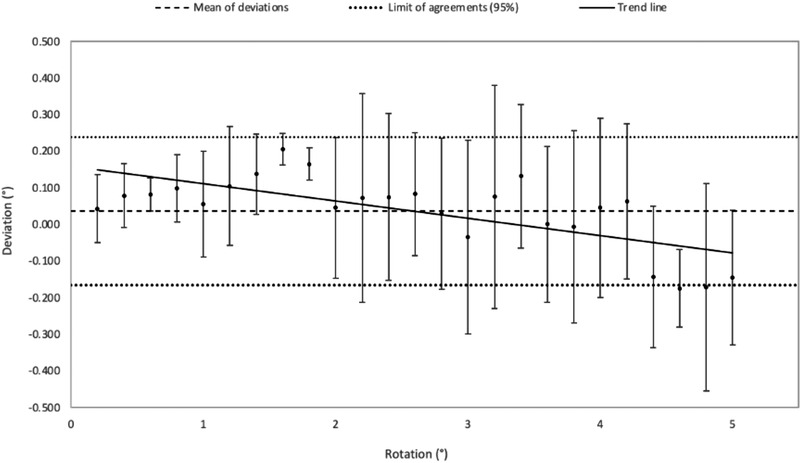
Bland–Altman graph representing the mean of the differences and standard deviation, in degrees (*n* = 30), between the rotations simulated on the MRI patient data and the rotations operated by the registration software

Figures 4, 5, 6 correspond to Bland–Altman representations of the mean of the differences between the translations (Figures 4 and 5) and rotations (Figure 6) induced on the patient's MRI and those performed by the registration algorithm as well as the standard deviation (SD) and the agreement limits (95%).

Along z, the registration was reproducible with the same translation performed by the registration algorithm (SD = 0, see Figure 5). Table 1 shows the corresponding Bland–Altman analysis. The results show that the positioning error (95% agreement limit) induced by the registration algorithm was less than 1.2 mm in the right‐left direction (*x*) and the anterior‐posterior direction (*y*) and less than 0.27 mm in the superior‐inferior direction (*z*). For pitch and roll, this error was less than 0.36°, for yaw, it was less than 0.6°.

**TABLE 1:** Results of the Bland–Altman analysis associated with the accuracy of the image registration algorithm

	Mean of deviations	Standard deviation	Limit of agreement (superior)	Limit of agreement (inferior)
*x*,*y* (mm)	−0.48	0.35	0.22	−1.18
*z* (mm)	−0.19	0.04	−0.12	−0.27
Rotations (°)	0.04	0.1	0.24	−0.17

### 3.2 CT‐MRI registration shifts relative to the trimodality techniques

#### 3.2.1 Phantom study

**FIGURE 7:**
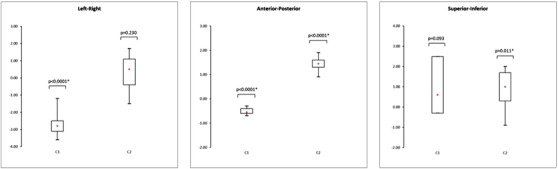
Box plots representing the translations (mm) performed on the CT‐MR images of the phantom by the registration algorithm with (C1) and without (C2) the transfer system (*n* = 10, * *p*‐value < 0.05)

**FIGURE 8:**
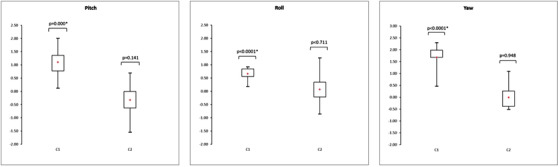
Box plots representing the rotations (°) performed on the CT‐MR images of the phantom by the registration algorithm with (C1) and without (C2) the transfer system (*n* = 10, * *p*‐value < 0.05)

Figures 7 and 8 show box plots representing the translations and rotations performed with the software to register the CT and MRI phantom images. The box plots represent the mean, minimum, maximum, interquartile range, and *p*‐value of the *t*‐test. The associated numerical results and the total error between CT and MR images are given Tables 2, 3, 4. For each translation and rotation, the box plot on the left represents the results of the ten tests performed using the transfer system (C1), and the box plot on the right represents the results of the ten tests performed without the transfer system (C2) by repositioning the phantom.

**TABLE 2:** Descriptive translation statistical analysis of the phantom positioning errors with (C1) or without (C2) the transfer system

**Translation (mm)**	Left‐Right (C1)	Left‐Right (C2)	Ant‐Post (C1)	Ant‐Post (C2)	Sup‐Inf (C1)	Sup‐Inf (C2)
Shift range	[−3.60;−1.20]	[−1.50;2]	[−0.70;0.30]	[0.90;1.90]	[−2.20;2.50]	[−0.90;2.10]
Mean	−2.70	0.34	−0.50	1.44	1.15	0.82
Median	−2.90	0.50	−0.50	1.45	0.90	1.40
Interquartile range	0.60	1.50	0.20	0.30	2.80	1.40

**TABLE 3:** Descriptive rotation statistical analysis of the phantom positioning errors with or without the transfer system

**Rotation (°)**	Pitch (C1)	Pitch (C2)	Roll (C1)	Roll (C2)	Yaw (C1)	Yaw (C2)
Shift range	[0.11;2.01]	[−1.55;0.69]	[0.17;0.92]	[−0.86;1.26]	[0.46;2.29]	[−0.52;1.09]
Mean	1.10	−0.33	0.66	0.07	1.68	−0.01
Median	1.12	−0.29	0.74	−0.14	1.86	−0.29
Interquartile range	0.59	0.63	0.29	0.56	0.30	0.65

**TABLE 4:** Total phantom positioning errors for translations and rotations

Translation	Left‐Right (C1)	Left‐Right (C2)	Ant‐Post (C1)	Ant‐Post (C2)	Sup‐Inf (C1)	Sup‐Inf (C2)
Total error (mm)	2.74	0.59	0.69	1.52	1.17	0.84
Rotation	Pitch (C1)	Pitch (C2)	Roll (C1)	Roll (C2)	Yaw (C1)	Yaw (C2)
Total error (°)	1.10	0.33	0.66	0.08	1.68	0.04

Regardless of the configuration used, the positioning error was higher than the error from the registration algorithm. Furthermore, there was no significant improvement in positioning accuracy when using the transfer system compared to when the phantom was completely repositioned between the CT and MR acquisitions. There was a systematic error in the translation, except in the superior‐inferior region, as well as in the rotation when the transfer system was used. This error was up to 3 mm for the translation and 1.7° for the rotation. The *t*‐test showed significant deviations from zero for the left‐right (*p* < 0.0001) and anterior‐posterior (*p* < 0.0001) translations of configuration C1. In terms of rotation, significant differences were observed for each angle. In configuration C2, there were only significant deviations from zero for the anterior‐posterior (*p* < 0.0001) and superior‐inferior (*p* = 0.011) translations.

#### 3.2.2 Patient study

**FIGURE 9:**
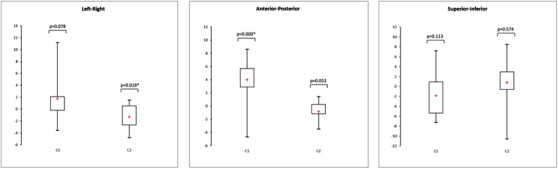
Box plots representing the translations (mm) performed on the CT‐MR images of the patient data by the registration algorithm with (C1) and without (C2) the transfer system (*n* = 15, * *p*‐value < 0.05)

**FIGURE 10:**
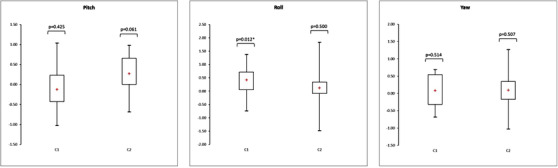
Box plots representing the rotations (°) performed on the CT‐MR images of the patient data by the registration algorithm with (C1) and without (C2) the transfer system (*n* = 15, * *p*‐value < 0.05)

Figures 9 and 10 show the box plots representing the translations and rotations performed by the software to register the CT and MRI patient data. For each translation and rotation, the left box plot represents the results of the 15 patients who were moved with the transfer system (C1), and the right box plot represents the results of the 15 patients repositioned on each imaging device without the transfer system (C2). These box plots represent the mean, minimum, maximum, interquartile range, and *p*‐value of the *t*‐test. The associated numerical results and the total error between CT and MR images are given Tables 5, 6, 7.

**TABLE 5:** Descriptive translation statistical analysis of the patient positioning errors with or without the transfer system

**Translation (mm)**	Left‐Right (C1)	Left‐Right (C2)	Ant‐Post (C1)	Ant‐Post (C2)	Sup‐Inf (C1)	Sup‐Inf (C2)
Shift range	[−3.60;11.20]	[−4.80;1.50]	[−4.70;8.60]	[−3.50;1.40]	[−7.30;7.20]	[−10.60;8.50]
Mean	1.75	−1.31	4.00	−0.83	−1.87	0.79
Median	1.20	−1.50	3.70	−0.70	−2.40	1.80
Interquartile range	2.30	3.20	2.80	1.40	6.30	3.55

**TABLE 6:** Descriptive rotation statistical analysis of the patient positioning errors with or without the transfer system

**Rotation (°)**	Pitch (C1)	Pitch (C2)	Roll (C1)	Roll (C2)	Yaw (C1)	Yaw (C2)
Shift range	[−1.03;1.03]	[−0.69;0.97]	[−0.74;1.37]	[−1.50;1.83]	[−0.69;0.69]	[−1.03;1.26]
Mean	−0.13	0.27	0.42	0.11	0.08	0.09
Median	−0.23	0.29	0.57	0.06	0.11	0.11
Interquartile range	0.66	0.66	0.66	0.43	0.86	0.52

**TABLE 7:** Total patient positioning errors for translations and rotations

Translation	Left‐Right (C1)	Left‐Right (C2)	Ant‐Post (C1)	Ant‐Post (C2)	Sup‐Inf (C1)	Sup‐Inf (C2)
Total error (mm)	1.81	1.39	4.03	0.96	1.88	0.81
Rotation	Pitch (C1)	Pitch (C2)	Roll (C1)	Roll (C2)	Yaw (C1)	Yaw (C2)
Total error (°)	0.14	0.27	0.42	0.12	0.09	0.1

Again, these results indicate no significant improvement in the positioning accuracy when using the transfer system compared to complete repositioning of the patient between the CT and MRI acquisitions. The *t*‐test showed significant deviations from zero for C1 for the anterior‐posterior and roll displacements, whereas for C2, there was only a significant deviation from zero for the left‐right displacements.

Similarly to the phantom study, the error related to the trimodality technique contributed the most to the total error.

## 4 DISCUSSION

In this study, the intrinsic positioning uncertainties of two PET/CT‐MR trimodal imaging configurations were evaluated. The first used an air‐cushion system to transport the patient from the PET/CT device to the MRI device, without changing the position between the two acquisitions (C1). The second configuration was a traditional radiotherapy setup. MRI acquisition was performed before the patient was repositioned under identical conditions for PET/CT acquisition (C2). In radiotherapy, accurate positioning during all treatment steps is essential. For instance, in the case of H&N cancers, this accuracy can reach 1–2 mm with adapted restraints such as thermoformed masks.^1^ Thus, any new process must be carefully evaluated before it is used in patient management during radiation therapy.

Our results show that the primary source of positioning uncertainty is the act of patient repositioning, regardless of the configuration. The uncertainty due to the image registration algorithm is small compared to that from the repositioning of the patient from one imaging device to another. Furthermore, patient positioning was not improved when the transfer system was used.

First, we verified that the registration algorithm was accurate with respect to the other sources of uncertainty. For the x and y translations, the algorithm's error was less than 1.2 mm for a range of induced offsets between 0 and 20 mm, and the uncertainty obtained along the z‐axis led to a positioning error of up to 0.27 mm. For the rotations, the largest error did not exceed 0.24° for angles between 0 and 5°. These results are in agreement with previous literature.^23^, ^28^ To simplify the results after verification, the analysis grouped the rotations and translations in the *xy*‐plane. However, it was not possible to group the translation along the z‐axis with translations in the *xy* plane.

Another source of uncertainty that was not addressed in this study is geometric distortions on MRI owing to the nonlinearity of gradients and internal or external field heterogeneities. Because these distortions are inherent to this technique, they may affect the multimodal registration.^29^ In future studies, it would be interesting to quantify these factors and evaluate their impact on registration.

The positioning uncertainties specific to the trimodality techniques were evaluated using a phantom. In this case, the uncertainties obtained were less than 3.6 mm in translation when the transfer system was used (C1) and less than 2.1 mm when the phantom was repositioned (C2). In terms of the rotations, the uncertainties were less than 2.3° for C1 and less than 1.5° for C2. In the C1 configuration, there were significant deviations from zero for both the translations and rotations. In the case of C2, there were only significant deviations from zero in the translations. Systematic errors were present when the transfer system was used, regardless of the translation and rotation directions, except along the z‐axis. Although the mask limited all movements, there was variability specific to the use of the transfer bed. The fact that it was an air‐cushioned system influenced the variability in the anterior‐posterior positioning and rotations because the system's compression could vary from one imager to another. In the right‐left and superior‐inferior directions, the positioning uncertainties were primarily due to the variability associated with the positioning of the Zephyr XL stage on each imager. The system's indexation made it impossible to precisely obtain the same position for each acquisition on each imaging device. The thermoformed mask was fixed on a flat tabletop, for which there was less variability in positioning on the examination table where the phantom was repositioned. Technical improvements in the transfer device could make it possible to eliminate shifts related to system indexation on the examination table and thus reduce the related positioning errors.

Finally, the positioning uncertainties were evaluated for both techniques in the two cohorts of patients under the clinical conditions of radiotherapy. There were significant deviations from zero for C1 for one translation axis and one angle of rotation. In the case of C2, there was only a significant difference for one translation axis. The radiotherapy mask minimized the translations and rotations, regardless of whether the transfer system was used. When using the transfer system, anterior posterior translations and rotations could be limited by the patient's weight, which reduced the variability associated with compression of the air‐cushioned system. There was little impact on patient movement in both cases. Apart from a few extreme values, the average translations obtained were between 0 and 4 mm, and all rotations were less than 1.8°. Despite this, from a clinical point of view, not using a transfer system is favorable to this solution in terms of user flexibility because the temporal synchronization of the two examinations is considerably less constraining. Moreover, an additional constraint for the patient is the total duration of the two examinations, during which they are unable to stand up.

The phantom study yielded worse results than the patient study, particularly for the three rotations with the C1 configuration. This is due to the fact that the patient has a larger size and weight than the phantom limiting rotation errors. In addition, the mask is designed to be used on patients rather than on the phantom. In particular, it fits the patient better than the phantom in the x and z directions.

This transfer system has also been evaluated in two previous studies using translation only. In the first,^24^ the authors found higher mean errors in 31 patients (abdomen, chest, and pelvis) than in our experiment, that is, 8.1 mm in *x*, 5 mm in *y*, and 4.9 mm in *z*. In the second study,^26^ in a cohort of 20 patients (pelvic cancer), the authors found errors of the same order of magnitude as those obtained in our experiment, except in anterior‐posterior, where we found a smaller range of values (0–4.8 mm in our study compared with 0–18 mm in their study).

## 5 CONCLUSION

This study showed that the transfer table used does not improve patient positioning in H&N cancer treatment with radiotherapy based on trimodality imaging. Nevertheless, it presents an interesting evolution requiring a careful technological development considering the risks of patient positioning errors between the two imaging devices. The accuracy of patient positioning must also be evaluated before these configurations can be used clinically for patients.

## AUTHOR CONTRIBUTIONS

P.H., I.G., P.G., P.D., H.S., P.V., and D.G. helped in conceptualization and project administration; P.H., P.G., P.D., S.T., and O.V. contributed to data curation; P.H. and I.G. wrote the main manuscript text and prepared the figures. All authors read and approved the final manuscript.
